# Epidemiologic characteristics of scrub typhus on Jeju Island

**DOI:** 10.4178/epih.e2017060r

**Published:** 2018-02-23

**Authors:** Sung Uk Lee

**Affiliations:** Jeju Special Self-Governing Provincial Office, Jeju, Korea

In the article [1], patient and disease characteristics were examined in 446 patients with scrub typhus that occurred in the Jeju area between 2011 and 2016. The most commonly observed history of exposure was fruit farming (n= 155, 35% of the entire sample), and 91% of those with an exposure to fruit farming worked to harvest citrus fruits. That is, a “phenomenon” was observed in which citrus farming is likely to be an important infection route of scrub typhus on the Jeju Island. It is stressed, however, that the study was not conducted with an aim to establish and prove a certain hypothesis with statistical techniques. Rather, the focus of the study was on quantifying the surveyed information to derive facts themselves. To “hypothesize” about the “phenomenon” observed in the present study, additional research should be conducted.

Below are responses to the problems additionally raised by the reader.

First, it is problematic since the age-adjusted and sex-adjusted incidences including 95% confidence intervals were not used. Figure 1 and Table 2 in the original manuscript [1] have been newly generated after calculating the age-adjusted incidence using the data from Statistics Korea in January 2016 (Table 1 and Figure 1) [2]. Please note that Figure 1 was obtained based on mean values of 6 years from 2011 to 2016. In comparison to the crude incidence in the original manuscript [1], there were clear number changes (mostly increases) in ‘eub’ and ‘myeon’ regions than ‘dong’ regions. Thus, we could observe the same trend as that in the original manuscript [1] by comparing both incidence rates by region and between regions.

The second is regarding the reader’s comment on the use of Mann-Whitney U-test in statistical analysis. The author of the present study aimed to examine whether the annual incidence rates per 100,000 population were significantly different between two independent areas, A and B, during the 6 years. Using Mann-Whitney U-test in such a case is also recommended in the reference cited by the reader. Because what the author wanted to find out was whether or not there were a regional difference between A and B, trend analysis was irrelevant in the present study.

The third is regarding the reader’s comment on Table 3. This table shows the distributions of cases by main cause of scrub typhus in each region. The table was intended to demonstrate that “the distribution was different across 6 regions,” rather than to show “prevalence odds ratios” with respect to risk factors. As discussed in the article [1], the table was presented to suggest that the focus of scrub typhus prevention projects should differ for each public health center, because the main causes were different among the regions. Also, the study found that the number of infected patients increased following citrus farming but unfortunately, it is not a study intended to prove that citrus farming is more risky than other types of farming.

Fourth, the relevance of high incidence in 2016 to the mean temperature and dryness in August was not an argument made by the author. The author simply cited the inference made by the Korea Centers for Disease Control and Prevention [3].

With these, the author expresses his thanks for the interest in the present study and the work of the reader and closes this reply.

## Figures and Tables

**Figure 1.**
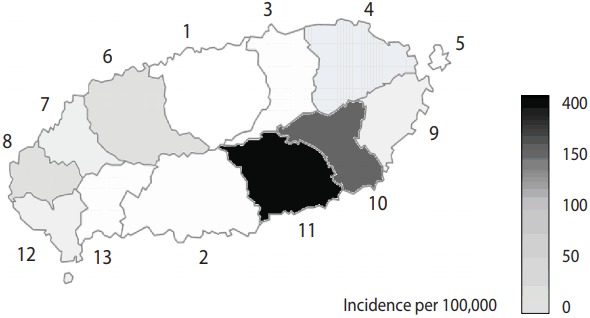
Average age-adjusted incidence rate per 100,000 of scrub typhus in Jeju Island, 2011-2016 (1, Jeju-si;2, Seogwipo-si; 3, Jocheoneup; 4, Gujwa-eup; 5, Udo-myeon; 6, Aewol-eup; 7, Hallim-eup; 8, Hangyeong-myeon; 9, Seongsan-eup;10, Pyoseon-myeon; 11, Namwon-eup; 12, Daejeong-eup; 13, Andeok-myeon).

**Table 1.** Age-adjusted incidence rate^1^ of scrub typhus per 100,000 by region in Jeju province

	Regions	2011		2012		2013		2014		2015		2016		p-value^2^
		Case (n)	Incidence	Case (n)	Incidence	Case (n)	Incidence	Case (n)	Incidence	Case (n)	Incidence	Case (n)	Incidence	
City	Jeju-si	9	0.7	16	2.0	13	1.6	14	1.9	6	0.3	34	4.0	0.004
	Seogwipo-si	12	18.0	10	10.7	8	8.8	9	10.5	13	19.8	16	12.2	
Country-side	Jeju East	1	0.7	7	28.8	8	49.4	4	10.8	7	29.6	10	25.3	0.20
	Jeju West	10	28.2	13	40.9	8	20.2	11	33.8	18	80.8	21	45.9	
	Seogwipo East	18	152.5	18	130.4	13	89.6	16	120.7	13	71.5	60	629.3	0.004
	Seogwipo West	7	57.9	8	64.7	2	5.3	1	1.2	4	16.7	8	27.8	

1 Population distribution data by age was applied as of January 2016 for estimating age-adjusted incidence.

2 p-value by Mann–Whitney U-test for the incidence.
