# Supplementary material for: Epidemiologic characteristics of scrub typhus on Jeju Island
Source: Epidemiol Health. 2018 Feb 23;39:e2017060r. doi: 10.4178/epih.e2017060r (PMC5847968; doi:10.4178/epih.e2017060r)
Supplement: Supplementary file 1 [file epih-39-e2017060r-supplementary.pdf]

Volume: 39, Article ID: e2017060r
https://doi.org/10.4178/epih.e2017060r

저자 회신

Epidemiologic characteristics of scrub typhus on Jeju Island

Sung Uk Lee
Jeju Special Self-Governing Provincial Office, Jeju, Korea

이 논문[1]은 2011년부터 2016년까지 제주 지역에서 발생한 쯔쯔가무시증 환자 446명에 인적, 질병적특성에 대한 분석을 시행하였다. 전체 환자 중 쯔쯔가무시증에 감염된 가장 흔한 노출력이 과수업 (155명, 전체 35%), 이중 91%가 감귤 수확 작업이었다. 즉 제주도에서는 감귤 과수원 농업이 쯔쯔가무시 감염의 주요 추정감염경로라는 '현상'이 관찰되었다. 하지만 본 연구는 통계기법으로 어떠한 가설을 추론하고 증명하려는 연구가 아니라, 조사된 정보들을 수치화하여 사실 자체를 드러내는데 주안점이 맞춰져 있다. 본 연구에서 관찰된 '현상'을 '가설'화 하여 증명하려면 추가적인 연구가 필요할 것이다.

독자께서 제기 하신 지적사항에 대한 답변은 다음과 같다.
첫 번째, 95% 신뢰구간을 포함하는 age and sex-adjusted incidence 를 사용하지 않았기 때문에 문제가 있다는 지적이다. 2016년 1월 통계청 자료[2]를 이용하여 age-adjusted incidence 구하여 원문의 Figure 1과 Table 2를 새롭게 구성하여 보았다(Table 1 and Figure 1). 단 Figure 1는 2011년부터 2016년까지의 6년 평균을 구하였다. 원문의 crude incidence와 비교해 보았을 때, 동 지역보다는 읍면 지역의 수치 변화 (주로 증가) 가 두드러졌다. 지역별 발생률의 경향과 지역간 비교에서 원문과 동일한 경향을 관찰할 수 있었다.

두 번째, 통계 기법 중 Mann-Whitney U-test 적용에 대한 지적이다. 저자는 독립된 A, B지역간에 6개 연도 각각의 10만명당 발생률 값이 의미 있는 차이가 있는지 보려고 하였다. trend를 보려는 목적으로 사용한 것은 아니었다

세 번째, 표3은 6개 지역 별 쯔쯔가무시증의 주요 원인의 case별 분포를 나타낸 표이다. 본 표에서는 위험요인에 대하여 'prevalence odds ratio'를 보기 보다는 단순히 '6개 지역별 분포가 다름' 만 나타내려 하였다. 본문에 기술하였듯이 6개 지역별 주요 원인이 다르므로 지역 보건소별 쯔쯔가무시 예방 사업의 포커스가 달라져야 함을 보여준 표이다. 또한 본 연구는 감귤농사 후 감염된 환자가 많다는 것이지, 아쉽게도 감귤농사가 다른 농사보다 더 위험함을 증명할 수 있는 연구가 아니다.

네 번째, 2016년도 발생사례가 높은 것이 8월의 평균기온 및 습도와 관련 있다는 것은 본 연구의 주장이 아니라 질병관리본부[3]에서 추정한 내용을 인용한 것이다.

Volume: 39, Article ID: e2017060r
https://doi.org/10.4178/epih.e2017060r

이상으로 독자께서 본 연구에 보여주신 관심과 노고에 감사함을 표하며 답신을 마무리하고자 한다.

REFERENCES

1. Lee SU. Epidemiologic characteristics of scrub typhus on Jeju Island. Epidemiol Health 2017;39:e2017039.
2. Statistics Korea. 'Demographic change in Jeju province'; 2016 [cited 2017 Dec 31]. Available from: m.kostat.go.kr/board/file_dn.jsp?aSeq=354804&ord=1 (Korean).
3. Korea Centers for Disease Control and Prevention. Infectious disease surveillance year book 2016; 2017 [cited 2017 Aug 1]. Available from: http://cdc.go.kr/CDC/info/CdcKrInfo0302.jsp?menuIds=HOME001-MNU1132-MNU1138-MNU0038&fid=32&q_type=&q_value=&cid=75290&pageNum=(Korean).
